# Prevalence and Predictors of Blood Pressure Screening in Karachi: A Cross-sectional Study

**DOI:** 10.7759/cureus.2967

**Published:** 2018-07-11

**Authors:** Marium Mehmood, Aleena Mesiah, Fizza Z Raza, Zainab Junaid, Munira Jamali, Jabeen Zehra, Syeda A Johar, Mahin Fatima, Hooria Imran, Fabeha Zafar, Darab Shaban, Fahad Amin, Duaa Rao, Faryal Khan, Emaan Amin, Naveen Tariq, Kaneez Fatima

**Affiliations:** 1 Department of Internal Medicine, Dow University of Health Sciences (DUHS), Karachi, PAK; 2 Internal Medicine, Dow University of Health Sciences (DUHS), Karachi, PAK; 3 Student, Dow University of Health Sciences (DUHS), Karachi, PAK; 4 Department of Internal Medicine, Dow Medical College, Karachi, PAK; 5 Department of Internal Medicine, Dow University of Health Sciences/civil Hospital Karachi, Karachi, PAK; 6 Department of Internal Medicine, Dow University of Health Sciences (DUHS), Karachi, PAK

**Keywords:** blood pressure screening, prevalence

## Abstract

Background

There is a lack of data about hypertension screening in low- to middle-income countries. The primary objective of this study was to determine the prevalence and predictors of blood pressure (BP) screening in Karachi, Pakistan. The secondary objective was to identify ways to improve effective BP screening practices among the population at risk.

Methods

This cross-sectional study was conducted from November 2016 to May 2017. The sample population consisted of 2039 residents of Karachi who were older than 18 years. A well-composed questionnaire was pilot tested and then used to assess their socio-demographic characteristics, personal attitude towards a healthy lifestyle, dietary habits, and BP screening practices. We used a chi-squared test as the primary statistical test.

Results

Of 2039 people, 1627 had their BP checked at least once in their lifetime. Approximately, half of the participants had their BP checked on a yearly basis. Women had a higher rate (83.6%, n = 989) of getting their BP checked than men (74.5%, n = 636). A significant relationship was observed between BP screening and lifestyle practices such as physical activity (p = 0.00), hours of sleep (p = 0.01), water intake (p = 0.01), and dining out (p = 0.03).

Conclusion

Current BP screening practices are inadequate amongst the urban population of Karachi. There is an urgent need for federal implementation of BP screening as well as awareness programs across the nation.

## Introduction

High blood pressure (BP) is one of the leading risk factors for global disease burden, accounting for 17% of all deaths worldwide [1]. The approximate number of adults with elevated BP has increased from 594 million in 1975 to 1.13 billion in 2015, 258 million of whom were living in South Asian countries [2]. The number of affected individuals is predicted to increase to a total 1.56 billion globally by 2025 [3]. Hypertension, a silent killer, is responsible for at least 45% of all deaths due to heart disease and 51% of deaths due to stroke [4].

In low- and middle-income countries where health systems are weak, the social predictors of health such as income, education, and housing adversely influence the development of hypertension and without effective intervention, there is a high probability that the rapidly growing burden of hypertension will worsen the global epidemic of cardiovascular and kidney diseases [5,6]. There is strong evidence that screening for treatment of high BP in adults substantially reduces the risk of cardiovascular events [7]. The major bottlenecks for effective hypertension control in middle-income countries are screening and effective titration of treatment [8].

Pakistan is ranked as the third country in the South Asian Association for Regional Cooperation for its high prevalence of hypertension. This could be attributed to population growth, aging and the presence of behavioral risk factors such as unhealthy diet, lack of physical activity, excess weight, and persistent stress [9]. Screening for high BP has been reported to be alarmingly low (35.6%) among the population of Pakistan because the country does not have a platform for detecting and treating high BP [10].

Though the prevalence of BP screening has been previously reported, little information has been compiled for its prevalence in Karachi. As it is the most populous city in Pakistan, Karachi contributes a major share to the national burden of hypertension. Accurate estimates of the prevalence of BP screening are essential as a source of primary information for the rational planning of health services, especially in a country with a weak healthcare system. Knowing the prevalence would allow the public health policymakers to assign sufficient priority to the management and prevention of hypertension. Considering the paucity of data, the primary objective of this study was to determine the prevalence and predictors of BP screening in Karachi. The secondary objective was to identify ways to improve effective BP screening practices among the population at risk.

## Materials and methods

After approval from the institutional review board of Dow University of Health Sciences, this cross-sectional study was conducted in Karachi from November 2016 to May 2017. The study targeted residents of Karachi older than 18 years who were currently living in Karachi and planned to be for more than a year. A sample size of 2,100 was calculated using a 95% confidence interval. A well-composed questionnaire was constructed via pilot testing. As part of the pilot testing, the questionnaire was reviewed by two medical practitioners to remove any ambiguity and to enhance its specificity.

The questionnaire gathered details on socio-demographic characteristics, personal attitude towards lifestyle practices, dietary habits, and BP screening practices. The factors that were assessed included age, gender, body mass index (BMI), area of residence, ethnicity, marital status, education, occupation, socioeconomic status (SES), daily water intake, salt, oily or sugary food intake, hot and soft drinks intake, daily hours of sleep, tobacco use, hypertension, medication for hypertension, hypertensive family history, and other comorbidities. SES was judged based on the number of household material items they owned (e.g., a car, motorbike, television, and/or air conditioner).

BP screening was assessed on the basis of self-reported answers to the following questions: “Have you ever had your BP checked?”; “How frequently do you have your BP checked?”; “Where do you have your BP checked?”. These questions were chosen to examine prevalence, consistency, and authenticity, respectively. Hypertension was defined by a systolic BP > 140 mm Hg, diastolic BP > 90 mm Hg, or an individual taking any antihypertensive medications [11]. When asked about their hypertension status, individuals who had high BP (> 140/90 mm Hg) on more than two occasions when measured at home but never assessed by doctor or health professional for hypertension in clinic were defined as unaware [12]. Hypertensive family history was positive when any of either the parents or siblings were hypertensive [13].

The interviewers were well-trained, and a standard protocol was conducted throughout to remove any interviewer bias. Additionally, the interviewers were also randomly checked by the principal investigators. The questionnaire was translated into Urdu to avoid any education bias. Both oral and written consent was obtained from participants. Anonymous data were collected voluntarily with no incentives offered. The cooperation rate was > 90%. A possible explanation for a co-operation rate higher than normally expected could be that the questionnaire was short, easily comprehensible and took an average of five to six minutes to be filled. No imputation methods were applied.

Data were analyzed using Statistical Package for Social Sciences (SPSS) version 20.0 (IBM Corp., Armonk, NY). Categorical variables were expressed using frequencies and percentages. Several continuous variables, including age, BMI, hours of sleep, and water intake, were also transformed into categorical variables to explore their relationship with BP screening in detail. A chi-squared test with a 95% confidence interval was used to compare categorical variables. In all cases, P values < 0.05 were considered statistically significant.

## Results

Of a total of 2,100 people approached for the study, 2,039 consented to be a part of the survey, giving a response rate of 97.1%. Most of the respondents were undergraduates (48.9%, n = 997), female (58%, n = 1130), single (55.9%, n = 1140), unemployed (56.5%, n = 1152), and from the middle SES (80.5%, n = 1641) (Table 1).

**Table 1:** Comparison of characteristics of people who were screened for BP with those who did not get screened for BP. BMI: Body mass index; BP: Blood pressure; CKD: Chronic kidney disease; CVD: Cardiovascular disease; DHA: Defence Housing Authority; DM: Diabetes mellitus; FB: Federal B.; PECHS: Pakistan Employees Cooperative Housing Society; SES: Socioeconomic status.

Social demographic characteristics	Total (%)		Screened for BP (%)		P Value	
		Yes		No		
Gender						
Male	42.0	74.5		25.5		.01
Female	58.0	83.6		16.4		
Age range (years)						
19-29	57.9	77.4		22.6		.01
30-39	12.4	77.3		22.7		
40-49	11.1	84.2		15.8		
50-59	10.8	86.3		13.7		
60+	7.8	88.6		11.4		
Marital status						
Married	44.1	83.4		16.6		.00
Single	55.9	77.0		23.0		
Education status						
Uneducated	4.1	71.4		28.6		.01
Primary	6.0	73.0		27.0		
Secondary	14.9	68.3		31.7		
Undergraduate	48.9	93.4		6.6		
Postgraduate	26.1	82.6		17.4		
Employment status						
Employed	43.5	78.7		21.3		.29
Unemployed	56.5	80.6		19.4		
SES						
Upper	11.5	82.1		17.9		.01
Middle	80.5	81.0		19.0		
Lower	8.0	66.3		33.7		
Comorbidities						
DM	9.1	91.3		8.7		.01
CVD	3.1	92.1		7.9		
CKD	1.8	83.7		16.3		
Hyperlipidemia	2.6	94.4		5.6		
Others	3.5	80.6		19.4		
None	79.9	77.4		22.6		
Residential area						
DHA Cantt	9.6	87.2		12.8		.01
FB Area	8.2	77.2		22.8		
Gulshan	25.6	82.5		17.5		
Korangi	3.6	78.1		21.9		
Malir	16.1	78.4		21.6		
North Khi	18.0	79.1		20.9		
PECHS	8.5	82.1		17.9		
Saddar	6.6	83.0		17.0		
Others	3.8	48.0		52.0		
Ethnicity						
Punjabi	12.2	84.0		16.0		.00
Sindhi	17.0	78.9		21.1		
Balouchi	3.2	63.1		36.9		
Pathan	5.4	70.3		29.7		
Urdu speaking	45.3	81.3		18.7		
Memon	3.0	77.4		22.6		
Other	13.9	80.8		19.2		
BMI (kg/m^2^)						
Underweight	17.8	72.7		27.3		.01
Normal weight	49.8	81.0		19.0		
Pre-obesity	22.2	81.7		18.3		
Obesity class 1	7.8	83.7		16.3		
Obesity class 2	1.3	85.2		14.8		
Obesity class 3	1.1	76.2		23.8		
Family history of hypertension						
Yes	54.6	86.2		13.8		.01
No	45.4	73.5		26.5		

About a quarter of the respondents (25.6%, n = 522) resided in the Gulshan area of Karachi, and approximately half (45.3%, n = 924) of the respondents were of Urdu-speaking ethnicity. Participants had a mean age of 33 years, and the most prevalent survey age group was 19 to 29 years old (57.9%, n = 1181). About half (49.8%, n = 1015) of the respondents had a normal BMI (Table 1).

Of the 2,039 participants, 1,627 had their BP checked at least once in their lifetime. Approximately half of the participants (n = 730, 45.0%) had their BP checked on a yearly basis. Almost half of the respondents (n = 1050, 51.5%) preferred to have their BP checked at home rather than at a clinical facility (Table 2). Women had a higher rate (83.6%, n = 989) of getting their BP checked than men (74.5%, n = 636) (Table 1).

**Table 2:** The prevalence of BP screening practices and their associations. BP: Blood pressure.

BP screening practices	Response (%)
Ever had your BP checked?	
Yes	79.8
No	20.2
Where was your BP checked?	
Home	51.5
Clinical facility	48.5
Frequency of BP checks	
Daily	3.5
Weekly	11.7
Monthly	39.8
Yearly	45.0
Hypertensive	
Yes	20.1
No	68.0
Unaware	11.9
Hypertension medications	
Yes	67.0
No	33.0
Is hypertension a systemic disease?	
Yes	72.7
No	14.9
I don’t know	12.4

A significant relationship was indicated between BP screening and age (P = .01), gender (P = .01), BMI (P = .01), marital status (P = .00), ethnicity (P = .00), educational level (P = .01), SES (P = .01), and residential area (P = .01). However, no association was observed between BP screening prevalence with employment status (P = .29). Roughly three-quarters (n = 1259, 77.4%) of participants who were screened had no other comorbidities.

Respondents who were aware that high BP can lead to other comorbidities were more likely to be screened (P = .01). Overall, 410 of 2,039 (20.1%) participants were hypertensive. Of these 410 participants, approximately two-thirds (n = 215, 67.0%) had been treated with antihypertensive medications. A participant having a family history of hypertension had a significant relation (P = .01) with BP screening prevalence.

A significant relationship was observed between the prevalence of BP screening and lifestyle practices such as physical activity (P = .00), hours of sleep (P = .01), water intake (P = .01), and dining out (P = .03). Respondents who slept more than 11 hours daily were the least likely to have their BP checked (58.3%, n = 28). Respondents who slept five to six hours or seven to eight hours were more likely to have their BP checked (81.1%, n = 472; 81.8%, n = 877) (Table 3).

**Table 3:** The prevalence of BP screening in relation to individuals’ attitude towards lifestyles. BP: Blood pressure.

Attitude towards lifestyle	Responses (%)	Screening for BP (%)			P Value
			Yes	No	
Tobacco use					
Yes	16.9		72.7	27.3	.01
No	83.1		81.6	18.4	
Physical activity					
Daily	33.0		76.6	23.4	.00
Weekly	25.6		84.0	16.0	
Monthly	17.8		83.0	17.0	
Never	23.6		78.5	21.5	
Hours of sleep					
Two to four	2.7		76.8	23.2	.01
Five to six	29.0		81.1	18.9	
Seven to eight	54.0		81.8	18.2	
Nine to ten	12.6		74.7	25.3	
Greater than ten	1.7		58.3	41.7	
Water intake/ (glasses per day)					
One to two	3.4		76.4	23.6	.01
Three to four	20.7		85.1	14.9	
Five to six	34.9		81.7	18.3	
Seven to eight	26.0		80.1	19.9	
Greater than eight	15.0		70.2	29.8	
Dine-out					
Daily	6.9		70.0	30.0	.03
Weekly	29.1		78.7	21.3	
Monthly	51.8		82.2	17.8	
Never	12.2		78.6	21.4	

Table 4 shows dietary habits in relation to the frequency of BP screening. No significant association was found between the incidence of BP screening and dietary habits such as the intake of fruits and vegetables, salty foods, sugary foods, oily foods, and hot drinks consumption. However, a significant correlation was seen between soft drinks consumption and BP screening (P = .01).

**Table 4:** Dietary habits of population screened for BP (79.9%, n = 1627). BP: Blood pressure.

Dietary habits	Response (%)			
	Daily (%)	Weekly (%)	Monthly (%)	Never (%)
Soft drinks	11.5	30.4	40.3	17.8
Hot drinks	81.7	8.4	4.6	5.3
Oily food	46.4	34.7	13.7	5.2
Sugary food	35.9	37.6	19.6	6.9
Salty food	32.8	37.3	22.6	7.3
Fruit and vegetables	64.2	29.5	5.2	1.1

## Discussion

We found that 74.5% of men and 83.6% of women had their BP checked at least once in their lifetime, with an overall prevalence of BP screening of 79.8%. The major predictors of BP screening were awareness regarding the associated risks and complications of high BP, a family history of hypertension, education level, SES, age, and gender. Another study previously conducted in Pakistan regarding BP screening found prevalence rates of 35.6% (41.3% for women vs. 9.0% for men) [10]. Most of the literature suggests that men have a higher prevalence of hypertension in comparison to women [14]. However, screening rates in this study were higher for women than men. This correlates with a study that found an underrepresentation of women in clinical trials performed on patients with hypertension. Subsequently, doctors tend to recommend more to men than women to have their BP checked [15].

In addition, most screened participants were undergraduates or postgraduates that belonged to a higher SES group. Thus, the most important predictors of screening for hypertension according to these results are literacy level and SES. To be screened, it is important for people to have awareness as well as the funds for routine visits to the clinic. We found that participants who were aware that hypertension can lead to comorbid diseases were more likely to be screened. It is likely that participants resent other comorbidities and are more prone to have their BP evaluated, possibly explaining the differences in comorbidities among measured and not. Furthermore, a family history of hypertension was also a major screening predictor; this supports the statement that those who were aware of the consequences of hypertension were more likely to have their BP checked.

Higher rates for screening were found in patients with known comorbid conditions. This comes as no surprise since diseases such as diabetes require regular follow-ups and having one’s BP checked is an essential part of a general physical examination [16]. The majority of those who were screened tended to have healthy lifestyle practices such as dining out less and better sleep habits. In recent years, there has been an uptick in general fitness awareness in urban Pakistani communities. This trend was reflected in the results of our study: of those who were screened 74.7% slept for nine to 10 hours and 81.8% had never smoked. Healthy lifestyle practices are also evident from the fact that half of those who were screened had a normal BMI and no other comorbidities. The majority dined out only monthly.

Nevertheless, the prevalence of BP screening in Karachi falls short of the ideal. As recommended by the U.S. Preventive Services Task Force, every individual aged 18 and older should be screened for high BP with additional measurements made for diagnostic confirmation [17]. Currently in the United States, about 89.6% of women receive this recommended screening, a percentage decidedly higher than the one in this study [18]. With hypertension being the most common risk factor for cardiovascular disease and incidence rates of hypertension being as high as 33% (in adults over 45 years) and 18% (in adults over 18 years), Pakistan is in dire need of more widespread screening for hypertension [19]. Around 70% of patients diagnosed with hypertension are unaware that it is a disease [20]. Because hypertension has no overt signs and symptoms and is only detected on screening, high BP remains the most common chronic condition diagnosed during outpatient visits [21]. Through indirect evidence, it has been shown that regular BP screening has substantial benefits; early detection is followed by early treatment and control. With proper therapy, the arrest of disease progression can be obtained, thus reducing mortality [22, 23]. The evolving socio-economic conditions of Pakistan include dietary trends shifting towards more fast food intake as well as increased use of oral contraceptives in women, paving the way for higher incidence rates of hypertension.

There is an acute need for nationwide health screening programs that include BP screening. Emphasis should be made for healthcare workers to follow the standard protocol of screening for BP abnormalities at every clinic visit regardless of the presenting illness. Awareness programs launched through mass media channels such as talk shows and television advertisements, highlighting the importance and need for hypertension screening, treatment, and control, should be introduced. Regular home measurements of BP should be encouraged by doctors. The prospect of public kiosks for self-measurement of BP can also be explored. Individuals could also benefit from randomized controlled trials studying the preventive effects of screening for hypertension (early detection) on cardiovascular mortality and morbidity. Further research is also needed to expand upon the recommended guidelines and protocols for BP measurement methods and screening intervals to reduce cases of misdiagnosis and ineffective screening.

There are several study limitations to our study. Convenience sampling was used to reach the target population. Due to this, the results may have suffered a degree of bias as the targeted population was not completely representative of the socioeconomic diversity of Karachi and were mostly in 19- to 29-year-old age group; this may have caused either under- or over-estimation of prevalence rates. Also, as a study based and conducted in Karachi, this work cannot be generalized to the broader population of Pakistan. Household monthly income may have been a more accurate indicator of SES than ownership of household items; however, many patients did not report it during the pilot study as they did not feel comfortable. Therefore, this option was changed to ownership of household items in the questionnaire. The data also lacked information regarding methods of BP measurement—this can also play a role in the accuracy of the diagnosis of hypertension. Nevertheless, this study is the only one to date to report the prevalence rates of BP screening in the general population of Karachi.

## Conclusions

The prevalence of BP screening found in Karachi is substandard. As an issue with a large room for improvement and exceedingly far-reaching health benefits, it is imperative that both the public and private sector be involved in working to maximize BP screening rates. The main reason for the low prevalence rates appears to be a lack of awareness. Owing to its silent nature, people tend to underestimate the many ways hypertension targets the body. Further studies are required to investigate BP screening awareness and practice across the country. The government should launch awareness programs that aim to rectify this gap in knowledge at a grassroots level. There is also an imminent need for doctors to educate the general population about the importance of at-home BP measurements.
